# Access to and value of information to support good practice for staff in Kenyan hospitals

**DOI:** 10.3402/gha.v8.26559

**Published:** 2015-05-14

**Authors:** Naomi Muinga, Barbara Sen, Philip Ayieko, Jim Todd, Mike English

**Affiliations:** 1 Department of Public Health Research, KEMRI-Wellcome Trust Research Programme, Nairobi, Kenya; 2 Information School, University of Sheffield, Sheffield, United Kingdom; 3 Department of Population Health, London School of Hygiene and Tropical Medicine, London, United Kingdom; 4 Department of Paediatrics, University of Oxford, Oxford, United Kingdom; 5 Nuffield Department of Medicine, University of Oxford, Oxford, United Kingdom

**Keywords:** health information, information needs of health workers, ICT, information sources, information seeking

## Abstract

**Background:**

Studies have sought to define information needs of health workers within very specific settings or projects. Lacking in the literature is how hospitals in low-income settings are able to meet the information needs of their staff and the use of information communication technologies (ICT) in day-to-day information searching.

**Objective:**

The study aimed to explore where professionals in Kenyan hospitals turn to for work-related information in their day-to-day work. Additionally, it examined what existing solutions are provided by hospitals with regard to provision of best practice care. Lastly, the study explored the use of ICT in information searching.

**Design:**

Data for this study were collected in July 2012. Self-administered questionnaires (SAQs) were distributed across 22 study hospitals with an aim to get a response from 34 health workers per hospital.

**Results:**

SAQs were collected from 657 health workers. The most popular sources of information to guide work were fellow health workers and printed guidelines while the least popular were scientific journals. Of value to health workers were: national treatment policies, new research findings, regular reports from surveillance data, information on costs of services and information on their performance of routine clinical tasks; however, hospitals only partially met these needs. Barriers to accessing information sources included: ‘not available/difficult to get’ and ‘difficult to understand’. ICT use for information seeking was reported and with demographic specific differences noted from the multivariate logistic regression model; nurses compared to medical doctors and older workers were less likely to use ICT for health information searching. Barriers to accessing Internet were identified as: high costs and the lack of the service at home or at work.

**Conclusions:**

Hospitals need to provide appropriate information by improving information dissemination efforts and providing an enabling environment that allows health workers find the information they need for best practice.

Information needs of health workers have been studied within a variety of settings in both developed and developing countries. These needs vary across different sectors of a health system (1). Different studies have identified these needs in line with specific themes such as reproductive health (2, 3) and clinical decision making (4) while others focussed on the needs of either particular cadres of health workers such as nurses or community health workers (5–7) or more general service providers (1, 2, 8, 9). The use of information communication technologies (ICT) for information dissemination and seeking has also been assessed albeit as part of specialised projects (4, 7, 10–12). Poor uptake of guidelines and poor access to relevant and reliable information by health workers would ultimately lead to poor care (13). A study by Nzinga et al. (14) reveals that one of the reasons for poor uptake of guidelines is that dissemination of information/knowledge tools like guidelines is inadequate.

Formal attempts to develop simple clinical guidelines for health workers in Kenya (15) have tried using ‘push’ methods to share information, which are prone to decay of knowledge and loss of skill over time (16). Perhaps insufficient attention is however given to ‘pull’ solutions which take advantage of health workers’ efforts to look for information to meet their present need. Pakenham-Walsh suggests a needs-led approach ‘where the information is based on research, informed by evidence, enabled by technology, and organized by subject (where appropriate)—but fundamentally led by needs’ (13).

Determining the information needs of health workers requires understanding of: health workers’ knowledge and practice in relation to the care they give their patients (17), perceptions of information types and sources available (5–7, 10, 18) and the means by which they seek information (7, 12, 19). These studies have however been conducted within specific projects or specific cadres of the health workforce and do not shed much light on user preferences for different sources of information (20).

In Kenya, online resources have become more accessible with over 7 million Internet subscriptions as at June 2012 (21) and almost universal mobile phone ownership among health workers (11). Mapping out the use of ICT by health workers for their day-to-day work could therefore inform policy makers on additional communication channels that they can make use of to enhance sharing of up-to-date and relevant information (22). This study therefore sought to investigate the information needs and preferences of health workers in Kenyan public hospitals. The specific objectives were: 1) to investigate where health workers in the Kenyan public hospitals go to when they have a question specific to the provision of modern/best practice in health care; 2) to investigate whether health workers in Kenyan public hospitals use ICT while seeking information related to their work; and 3) to investigate what solutions are provided formally by the hospital in an effort to support modern/best practice health care among health professionals in Kenyan public hospitals.

## Methods

### The study

The study was implemented as part of a larger study undertaken by KEMRI-Wellcome Trust Research Programme in collaboration with the Ministry of Medical Services (MOMs) as a partner in the Health Services, Implementation Research and Clinical Excellence (SIRCLE) collaboration in Kenya in the month of June 2012. The study was conducted in 22 hospitals that provide internship training to young doctors. MOMs purposefully identified these with a view to appropriate national, geographic representation from a total of 40 such hospitals linked to aims to assess the quality of care given to patients (23, 24). The data reported here were collected using self-administered questionnaires (SAQs) that were distributed to health workers across the 22 study hospitals.

### Questionnaire design and testing

Questionnaire design drew on prior studies that conducted information needs assessments of health workers (6, 7, 18, 25) with additional questions formulated to meet the study objectives. The SAQ included closed questions with options on a five-point Likert scale or other appropriate choices and open-ended questions. It was then tested and re-tested on five clinicians not in the study and modified after considering the feedback given. A third round of questionnaire testing was done on a further six health workers before piloting in two hospitals not included in the study. At each point modifications were made to improve clarity and remove redundancy.

### Data collection

Data for this study were collected for all hospitals by 22 health workers (one drawn from each study hospital). These health workers had undergone a 1-week training session together that introduced them to the study aims, principles of good research and data quality and that allowed them to pilot use of tools in a non-study hospital. Following this training, health workers returned to their hospitals and had between 2 and 3 weeks to distribute and collect the SAQs (23). Together with the survey team leader they checked the SAQs for completeness on collection making efforts to obtain missing data where possible. Personal identification information, such as names, was not included in the questionnaire.

### Sample size

Care is predominantly provided in each of the clinical areas by nurses, clinicians, and non-clinician physicians, with support from laboratory and pharmacy staff. During the limited survey period, health workers on duty within clinical sites were the main target of sampling as random selection from a staff list was not deemed feasible. The aim was to collect four SAQs in the mother and child health clinic (MCH) and outpatient department (OPD) and five each from the paediatrics, nursery and maternity, surgery, and internal medicine clinical areas representing a mixture of clinicians and nurses. In addition, up to six more questionnaires were to be distributed to other cadres such as lab technologists, pharmacists, nutritionists, radiologists, and physiotherapists if available making a maximum sample of 34 per hospital. Achieving this sample size would have been more than sufficient to report a prevalence of 50% for binary responses to SAQ questions with a precision of±10%. Even allowing for a design effect of 2 due to the clustered nature of the data such precision would be achieved with a total of 211 respondents after adjusting for a 10% non-response rate. Achieving a sample of 34 per hospital would also allow for adequately powered exploratory analysis using regression modelling.

### Data entry, quality, and analysis

Study data from the SAQs were entered and managed using Research Electronic Data Capture (REDCap), a secure, web-based application designed to support data capture for research studies. (26). The data were single entered then checked for accuracy by doing a double entry for 10% of the records with 97% agreement observed. The data were analysed using STATA version 11 (StataCorp LP, College Station, TX). Overall proportions (for binary responses) were calculated for all respondents adjusting for clustering at the hospital level. Two approaches were employed where the five-point Likert scale was used; cluster adjusted mean scores were generated for choices available; and where appropriate, options on the scale were collapsed to give a proportion adjusted for clustering.

We hypothesised that there were associations between two markers of ICT use (use of a computer to search the Internet for work-related information and use of a mobile device/tablet to access information related to work) and age, gender, cadre, and years of experience after internship. These associations were explored using simple univariable analyses. The covariables in these univariable models that appeared significant (*p*<0.05) were explored further in multivariable logistic regression models (except years of work after internship as it was poorly answered) for computer and mobile device use. Additionally we carried out tests to ascertain whether clustering exists for the outcomes of interest. Where clustering existed it was adjusted for in the model.

### Ethics

Scientific and ethical approval for the study was obtained from the Kenya Medical Research Institute (KEMRI) National Scientific and Ethical Review Boards. The Ministry of Health also approved the study and the study was explained to hospital management teams who provided their assent prior to data collection.

## Results

A total of 657 (median=31, range per hospital=16–34) SAQs were returned from a possible 748 distributed to the health workers (for a response rate of 86%). The majority of respondents were nurses (64%) followed by clinical officers (21%), and medical officers (8%). Over 50% of the respondents were aged over 36 years. The largest respondent group was from the maternal–child health and outpatient clinics (161/628, 26%). The full details of the characteristics of the respondents are shown in Table 1.

**Table 1:** Summary of characteristics of 649 Kenyan health workers surveyed

	Gender			
	
	Male		Female	
		
	*n* (%)	95% CI^a^	*n* (%)	95% CI^a^
How old are you?				
Below 25	32 (16.0)	10.9–21.1	36 (8.1)	4.8–11.4
25–29 years	69 (34.5)	27.3–41.7	63 (14.2)	8.9–19.4
30–35 years	39 (19.5)	13.3–25.7	70 (15.7)	10.6–20.9
36–45 years	31 (15.5)	10.7–20.3	149 (33.5)	29.8–37.1
Over 45 years	29 (14.5)	9.1–19.9	127 (28.5)	22.2–34.9
Total	200		445	
Cadre				
Medical officers	37 (18.5)	11.8–25.2	18 (4.0)	1.7–6.3
Clinical officers	75 (37.5)	31.3–43.7	61 (13.6)	9.6–17.6
Nurses	56 (28.0)	21.2–34.8	356 (79.3)	74.1–84.5
Others	32 (16.0)	9.3–22.7	14 (3.1)	1.2–5.0
Total	200		449	
Current area where you mostly work in the hospital				
Paediatric	32 (16.9)	11.5–22.3	78 (18.1)	14.3–21.8
Labour ward	26 (13.8)	7.8–19.7	75 (17.4)	13.4–21.4
MCH/OPD	39 (20.6)	14.8–26.5	119 (27.5)	21.4–33.7
Surgery	44 (23.3)	16.8–29.8	60 (13.9)	9.9–17.8
Medicine	40 (21.2)	14.1–28.2	92 (21.3)	18–24.6
Others^b^	8 (4.2)	0.8–7.6	8 (1.9)	0.3–3.4
Total	189		432	

a Adjusted for clustering.

b Nurses (nurses, BSc. nurse, nurse intern/student), clinical officers (CO interns, COs, specialist COs), medical officers (MOs, MO interns, consultant/specialists), others (records officers, pharmacists, lab technicians, nutritionists, etc.).

### Information needs of health workers and information provision by hospitals

**Fig. 1:**
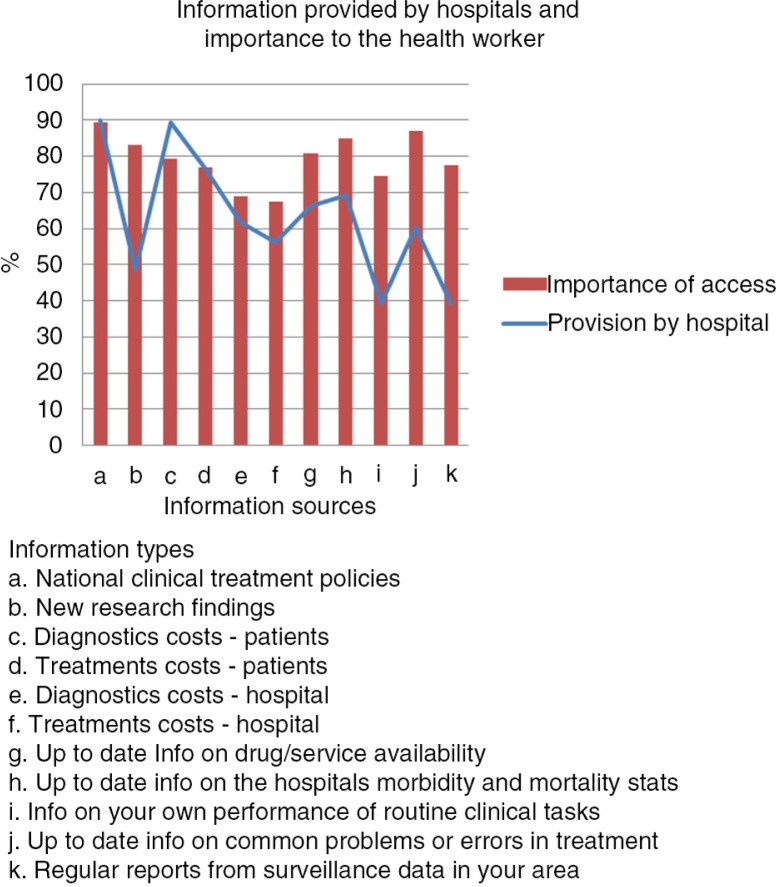
Information needs and provision of information by hospitals.

The health workers were asked to rate on a five-point scale how often in their day-to-day work (giving medical/nursing care) they felt the need to consult/get further information to help them do the right thing. Overall 70.4% responded that they commonly need additional information while giving care [score 4 and 5 (*N*=508, 95% CI=65.3–75.7%)] with no meaningful difference between cadres (data not shown). Types of information felt to be important and whether or not such information was supplied by the hospital were examined using a similar scale (1=not important at all to 5=very important). Responses were dichotomised (score 4 and 5 indicating important) prior to analysis. Figure. 1 shows the proportions of those who felt that different information types were important (score 4 and 5) and whether or not the hospital provided that information. National treatment policies and information on costs of services were provided by the hospitals (90%, *N*=644, 95% CI=87.1–93%, and 76.5%, *N*=620, 95% CI=71.5–81.4%, respectively) and considered important by a large majority of the health workers (89.4%, *N*=633, 95% CI=86.4–92.5%, and 76.9%, *N*=603, 95% CI=73.9–80%, respectively). On the other hand, there were information types that the health workers reported to be important to them (over 70% respondents) but provision by the hospital was lacking (reported as provided by fewer than 40% of respondents). These included: New research findings, information on health worker's performance of routine clinical tasks and regular reports from surveillance data (Fig. 1). The most common communication strategies used by hospitals to give information on best practice were one-to-one instruction and Continuous Medical Education (CME) sessions while email and text messages were rarely used (data not shown).

### Sources of information for health workers and barriers

Health workers were asked to rate on a five-point scale (least likely to very likely) where they were likely to seek further information to improve their assessment of patients or make the best diagnosis for each of six choices. Mean scores were calculated for each of the sources and indicate that; talking to colleagues at the same level was the most likely source of information to guide day-to-day practice (4.08, *N*=632, 95% CI=3.98–4.19) closely followed by talking to consultants (4.01, *N*=626, 95% CI=3.90–4.12) and printed guidelines (3.62, *N*=634, 95% CI=3.46–3.78). Turning to the Internet (3.13, *N*=632, 95% CI=2.98–3.27) or scientific journals (2.66, *N*=624, 95% CI=2.49–2.84) for information were least likely to be used.

Limited reliance on resources predominantly accessed via ICT was also observed when health workers were asked to rate how often they might use alternative reference resources (five-point scale: 1=Never to 5=Very often) to help improve their professional practice. Printed guidelines (3.75, *N*=497, 95% CI=3.62–3.88) and printed text books (3.41, *N*=489, 95% CI=3.22–3.59) were reported as most used with Internet resources less commonly used (3.11, *N*=481, 95% CI=2.93–3.28). Directly accessing journal databases (1.95, *N*=468, 95% CI=1.82–2.09) or electronic books (2.1, *N*=472, 95% CI=2.13–2.51) were much less common. The most common reason given for never using these poorly accessed sources was that they were ‘Not available/difficult to get’ and ‘Difficult to understand’.

### Use of ICT in information search by health workers

The use of ICT (computers and mobile phones) by health workers to find information for their day-to-day work was assessed on several aspects: access, use and barriers. The results show that 80.5% (*N*=614, 95% CI=73.8–87.1%) of health workers have access to a computer whether at home, work or at a cyber café and that 62.5% (*N*=602, 95% CI=55.3–69.6%) use a computer to search for work-related information from the Internet with 46.1% (185/401, 95% CI=39.7–52.6%) and 29.4%, (118/401, 95% CI=24.4–34.4%) conducting a search at least once weekly or daily. Across the different cadres usage of a computer to search the Internet for work-related information was lowest among nurses at 54.6% (*N*=379, 95% CI=45.4–63.9%). The most common barriers to using the Internet were: lack of access at home (39%, 95% CI=33.9–44%) or at work (36.8%, 95% CI=31.5–42.1%) followed by the high costs of accessing the Internet (34.1%, 95% CI=29.1–39.1%). About 19.8% (95% CI=14.7–24.9%) of the health workers reported to have no time to search for information on the Internet.

Ownership of mobile phones among the health workers was nearly universal (98.6%, *N*=639, 95% CI=97.5–99.7%) with 75.0% (*N*=633, 95% CI=70.9–79.2%) of health workers using their mobile devices to access work-related information. The most common uses of mobile phones for work were: calling colleagues to discuss a work-related question (60.3%, *N*=657, 95% CI=56.3–64.2%), sending text messages to answer or ask a question (57.5%, *N*=657, 95% CI=53–62.1%) and searching the Internet for further information for their work (55.9%, *N*=657, 95% CI=50.3–61.2%). Mobile phones were also used to post questions and receive responses from social networks, e.g. Facebook (25.1%, *N*=657, 95% CI=21.5–28.8%), and to access medical applications that the health workers might have downloaded (22.1%, *N*=657, 95% CI=17.6–24.7%). Across each of the different cadres, over 85% of the health workers reported having used a mobile device to access information related to their work. The usage among nurses was lower than other cadres at 67.3% (*N*=401, 95% CI=61.6–73%).

Results from the univariable analysis exploring the association between computer and mobile device use to search for work-related information, showed that there was a significant association with each of the characteristics (age, gender, cadre, years of work after internship). We then postulated potential interaction between age and gender, gender and cadre, and age and cadre and tested each logistic model with and without the interaction term for both ICT use outcomes. The likelihood-ratio tests showed no evidence for any interaction between the covariables. The likelihood-ratio test comparing the logistic regression models with and without clustering showed that there was evidence to indicate the presence of clustering at the hospital level (*p*=0.0002) for computer use, but little evidence for clustering at the hospital level for mobile phone use (*p*=0.207). The covariates in the univariable models were then added into multivariable logistic regression models for computer and mobile device use adjusted for clustering.

The results from the multivariable models show that Nurses were less likely to use a computer (OR=0.32; 95% CI=0.13–0.77; *p*=0.011) or mobile device (OR=0.18; 95% CI=0.06–0.55; *p*=0.003) to access information related to their work as compared to medical officers after adjusting for all the covariates (Table 2). Although there was insufficient evidence for a trend the results also suggested that health workers were less likely to use either a computer or mobile device to search for information as age increased with those aged 45 years and above significantly less likely to use a computer (OR=0.46; 95% CI=0.22–0.98; *p*=0.044) or mobile device (OR=0.28; 95% CI=0.11–0.74; *p*=0.010) to search for information than those aged below 25 years.

**Table 2:** Adjusted odds ratios, *p* values, and confidence intervals for use of ICT by health workers

	aUse a computer to search the Internet for information related to your work; *N*=590		Use of mobile device/tablet to access information related to your work; *N*=622	
		
Demographics	OR (95% CI)	*p*	OR(95% CI)	*p*
Age				
Below 25	1.00		1.00	
25–29 years	1.05 (0.5–2.18)	0.901	0.44 (0.16–1.18)	0.103
30–35 years	1.27 (0.58–2.79)	0.543	0.48 (0.18–1.30)	0.151
36–45 years	0.53 (0.25–1.11)	0.093	0.34 (0.13–0.88)	0.027
Over 45 years	0.46 (0.22–0.98)	0.044	0.28 (0.11–0.74)	0.010
Gender				
Male	1.00		1.00	
Female	0.85 (0.53–1.35)	0.495	1.15 (0.7–1.90)	0.577
Cadre				
Medical officers	1.00		1.00	
Clinical officers	0.42 (0.17–1.02)	0.056	0.47 (0.15–1.49)	0.198
Nurses	0.32 (0.13–0.77)	0.011	0.18 (0.06–0.55)	0.003
Others	0.68 (0.23–2.00)	0.485	0.48 (0.13–1.81)	0.281

a Odds ratios are adjusted for clustering.

## Discussion

Overall, health workers reported that they would need information in their day-to-day work to help them provide appropriate care. According to the health workers, national treatment policies and information on treatment costs for patients were provided by the hospitals and were found to be important to the health workers; this finding is consistent with literature (2, 5, 6, 20). Gaps in provision of new research findings and regular reports from surveillance data were identified however. Information on health worker's performance of routine clinical tasks was found be of value to health workers but not provided by hospitals. This information requires the presence of a good health information system that collects good quality data to feed into regular meetings by clinical teams to evaluate their performance, something currently lacking in this setting (27).

In common with previous studies, health workers reported that the most popular source of information was informal communication with colleagues and consultants (5, 6, 9, 28). Colleagues are likely to be ‘easy to access’ during routine activities as opposed to electronic or print media. This ease of access to information sources has been described as an attribute of useful medical information (29, 30). Colleagues have also been viewed as trusted sources of information (28) suggesting that such informal sources should not be ignored as dissemination channels. Scientific journals, journal databases and the Internet in general were rarely used by the health workers a finding that is similar to other studies (9). The most frequently cited barrier to sources that require adequate infrastructure (hardware and reliable and affordable access to the Internet) was that they were not available or difficult to use. Additionally, training and sometimes subscriptions are required to allow for their full potential to be realised.

The use of ICT in information seeking by health workers has been assessed as part of specialised projects in developing countries. The results show that health workers, who use electronic sources of information, make better decisions for their work and that mobile devices were used to access further information (10, 11, 19, 31, 32). The widespread ownership of mobile phones has been made possible by an enabling environment in Kenya where the last few years have seen an increase in availability of a range of mobile phones (both low cost and high end) but it is noteworthy that none of the hospitals studied provide free Internet access for frontline health workers. Internet costs have dropped due to competition among service providers; however some of the costs are still prohibitive for many. To put this into context, monthly basic salaries of health workers (using an exchange rate of 133 Kenyan shillings for 1 British pound in mid-2012) range from £232.65 to £418.80 for nurses and clinical officers and £268.09 to £489.68 for medical officers. The costs of browsing the Internet range from £0.52 to £0.59 per megabyte (MB). Although there are lower prices for bundles of data in pre-fixed MBs (as low as £0.04) this may still deter people from using data heavy sites.

Our data suggest that nurses, compared with medical officers, and those aged over 45 years were less likely to use ICT (computer and mobile device) for information searching; based on the findings of multivariable logistic regression model adjusted for demographic characteristics and clustering. This finding is perhaps not surprising as older individuals might not be as experienced in the use of technology for information seeking and are likely to be less frequent users of social media. Moreover, as one gets older, there may be more responsibilities financially and consequently accessing the Internet may become less of a priority. Conversely, it could be that uptake of ICT is more popular among the younger health workers and thus they are able to translate this use of technology to their work. This age effect may be important, however, as many nurses in rural areas are aged over 40 years in Kenya (33).

Clustering at the hospital level was found to be present for the outcome: use of a computer to search the Internet. This might be explained by recent efforts by hospitals to digitise operations, which in turn would help improve access to a computer with Internet access and subsequently access to online resources. A possibility supported by linked data on hospital infrastructure indicating that computer availability in the hospitals and their Internet connectivity varied considerably across hospitals (27).

### Limitations

The use of SAQs, while encouraging honest responses because of anonymity, limits the kind of data collected and does not allow for exploration of new ideas but does provide a starting point for more focussed work. The number of completed questionnaires received from various hospitals varied between 16 and 34 with a median response of 31 out of a possible 34 distributed to each hospital. The lower response rate in some hospitals might have been due to respondent fatigue as some health workers complained that there were too many surveys being conducted and no feedback on findings given. In addition, the varied numbers of responses and the length of the questionnaire might have contributed to poor completion by health workers. Lastly, the sample studied in this research comprised frontline health workers who interact directly with patients and are typically not involved in research activities. The consultants in the wards (e.g. Paediatricians) who may be classified as managers in the hospitals were not surveyed and these form a group that is likely to be different from the health workers surveyed hence their needs and sources might be different. It is therefore not possible to generalise these results to all health workers in internship hospitals in Kenya.

### Recommendations

The potential for the use of ICT as a tool to aid in knowledge/information propagation has not been fully harnessed. The Ministry of Health should explore possibilities of supporting hospitals that are providing initial experiential training to provide reliable access to the Internet by setting up resource centres and local area networks (LANs). Wireless connectivity can enable access to health workers through laptops and mobile phones while at work. In addition health workers should be trained on the use of online resources such as journal databases and the Internet and information searching in general. This should ideally promote the use of correct ideally pre-digested, summarised and locally relevant information found to be most relevant in a review by Revere (30). Additionally, hospitals should make use of communication means frequently used by health workers such as mobile phones to rapidly convey information for example on local surveillance findings or, in the future perhaps, performance feedback.

A central repository of such relevant and localised information for Kenyan health workers has the potential to increase dissemination of such materials, research findings and regular surveillance reports. LeMay and Bocock have shown that such central knowledge sharing efforts may overcome barriers to accessing up-to-date information (2).

## Conclusions

This study set out to find out where health professionals in internship hospitals in Kenya turn to for work-related information, which information is important to them and what solutions are provided by hospitals to meet these needs. Colleagues were a popular source of knowledge as opposed to published sources, from either academic journal or the Internet, possibly because they are easier to access, or because they genuinely trust their knowledge and expertise. The barriers identified reinforced this need for ease of access, and in this context this was largely due to problems with the information infrastructure, including access to computers. Further to this, demographic specific differences were noted with nurses being less likely to use ICT for health information searching, and older workers being less likely to use the Internet. Providing ICT solutions and meeting health worker demand for information would, however, seem to be an increasingly promising mechanism to address information gaps.
